# Impact of interval walking training managed through smart mobile devices on albuminuria and leptin/adiponectin ratio in patients with type 2 diabetes

**DOI:** 10.14814/phy2.14506

**Published:** 2020-07-11

**Authors:** Jelizaveta Sokolovska, Karina Ostrovska, Leonora Pahirko, Gunita Varblane, Ksenija Krilatiha, Austris Cirulnieks, Inese Folkmane, Valdis Pirags, Janis Valeinis, Aija Klavina, Leo Selavo

**Affiliations:** ^1^ Faculty of Medicine University of Latvia Riga Latvia; ^2^ Latvian Academy of Sport Education Riga Latvia; ^3^ Faculty of Physics, Mathematics and Optometry University of Latvia Riga Latvia; ^4^ Faculty of Computing University of Latvia Riga Latvia; ^5^ Centre of Nephrology Pauls Stradins University Hospital Riga Latvia; ^6^ Department of Internal Medicine Pauls Stradins University Hospital Riga Latvia

**Keywords:** albuminuria, diabetes, interval walking, leptin/adiponectin ratio, mobile application

## Abstract

**Background:**

Interval walking training has demonstrated more pronounced positive effects on physical fitness and metabolism in type 2 diabetes (T2D), compared to continuous walking. One of the pathogenic mechanisms of T2D is associated with derangements in leptin/adiponectin axis, which might predispose affected individuals to vascular inflammation and albuminuria. The aim of this study was to investigate the effects of interval walking training delivered through smart mobile devices upon albuminuria and leptin/adiponectin ratio in patients with T2D.

**Methods:**

Patients with T2D aged 35–75 were randomized into control (*n* = 26) and interval training (IT, *n* = 14) groups. Patients in IT group had to perform three 60‐min interval walking sessions (3 min intervals of slow and fast walking with the intensity of 40% and 70% of the peak energy expenditure) per week delivered by smartphone application for four months. The adherence to training was monitored remotely. Outcome measures were albuminuria, leptin/adiponectin ratio, obesity indicators, and glycaemic control. Leptin and adiponectin concentration was measured in serum samples by Luminex technology.

**Results:**

In the IT group compared to control group, we observed a statistically significant decrease in albuminuria (*p* = .002) and leptin/adiponectin ratio (*p* = .01), as well as a decrease in HbA1c close to statistical significance (*p* = .09). In IT group, changes in leptin/adiponectin ratio correlated significantly with changes in hip circumference (*p* = .024).

**Conclusion:**

Interval walking training is beneficial for vascular health in T2D via impact on albuminuria and leptin/adiponectin ratio.

## Graphical abstract

The central question of this study is weather interval walking training affects risk factors of vascular complications in diabetes. We found that in patients with type 2 diabetes who performed interval walking 3 times a week for 60 min and 4 months long, reduction of albuminuria and leptin/adiponectin ratio is observed in the absence of significant changes in glycaemia, body mass index and HOMA‐IR index

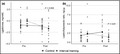

## 1 INTRODUCTION

Patients with type 2 diabetes are at an increased risk for development of vascular complications, such as myocardial infarction, stroke, peripheral vascular disease, retinopathy, neuropathy and nephropathy (Cosentino et al., 2019). Physical activity is the first‐line treatment in type 2 diabetes(Colberg et al., 2016; Pai et al., 2016). Recently, promising data about the positive impact of physical activity on the vascular complications of diabetes have emerged (Lin, Peng, Chiou, & Huang, 2014; Pongrac Barlovic, Tikkanen‐Dolenc, & Groop, 2019; Tikkanen‐Dolenc et al., 2017). Moreover, it has been shown that physical activity is associated with the reduction in albuminuria (Hellberg, Höglund, Svensson, & Clyne, 2019). Clinically significant albuminuria (microalbuminuria and macroalbuminuria) is a well‐known marker of endothelial damage and a prognostic marker for cardiovascular diseases and renal outcomes (Vyssoulis et al., 2010; de Zeeuw et al., 2004). However, higher albuminuria within the normal range has also been associated with adverse cardiovascular outcomes (Blecker et al., 2011).

The molecular mechanisms by which physical activity might prevent or slow down the development of vascular disease are under investigation nowadays. It is known that the hemodynamic effects of physical activity induce a variety of beneficial vascular adaptive processes (Green, Hopman, Padilla, Laughlin, & Thijssen, 2017). Taking into account the effect of physical activity on weight reduction and insulin sensitivity, adipocytokines might be among the effectors of physical activity on vasculature. In individuals with type 2 diabetes, adiponectin levels are decreased and leptin levels are increased due to leptin resistance. Derangement in leptin/adiponectin axis, which is characterized by increased leptin/adiponectin ratio in these conditions might predispose affected individuals to vascular inflammation and therefore the progression of micro‐ and macrovascular complications of diabetes (Katsiki, Mikhailidis, & Banach, 2018; Liang & Ye, 2019; Lim et al., 2015; López‐Jaramillo et al., 2014; Nosalski & Guzik, 2017; Rodríguez, Nunes, Mastronardi, Neeman, & Paz‐Filho, 2016). A recent meta‐analysis has shown that aerobic exercise increases adiponectin and reduces leptin levels in adults with prediabetes and diabetes (Becic, Studenik, & Hoffmann, 2018).

Interval walking training has demonstrated good tolerability and more pronounced positive effects on physical fitness and metabolism in type 2 diabetes, compared to continuous walking (Gibala, Little, MacDonald, & Hawley, 2012; Jelleyman et al., 2015; Karstoft et al., 2013; Morikawa et al., 2011). The personalized intensity during training can be achieved using smart phone mobile applications which need a measure of personal physical fitness (e.g. results of VO2 peak test or six‐minute walk test; Kuziemski, Słomiński, & Jassem, 2019) obtained before starting a new exercise plan. However, the effects of such training on markers of vascular health, including albuminuria and adipocytokine levels, have not been assessed.

Thus, the aim of this study was to investigate the effects of personalized interval walking training delivered through smart mobile devices on albuminuria and leptin/adiponectin ratio in patients with type 2 diabetes.

## 2 MATERIALS AND METHODS

### 2.1 Recruitment and screening

The study was performed in Riga, Latvia, at the Laboratory for Personalized medicine, Faculty of Medicine, University of Latvia, between October 2017‐September 2018. The study recruited 78 patients with type 2 diabetes via information disseminated by public media. Screening included medical history interview, physical examination (weight, height, waist and hip circumference, blood pressure, heart rate). Exercise stress electrocardiography (veloergometry) was performed by a certified cardiologist to identify patients with increased cardiovascular risk.

Inclusion criteria were: previously established type 2 diabetes (data from medical records), age of 35–75 years. Exclusion criteria were: results of the exercise stress electrocardiography corresponding to increased exercise‐induced cardiovascular risk, insulin therapy, regular structured physical activity (>150 min/week), pregnancy, pre‐existing cardiovascular disease (unstable angina, myocardial infarction, stent/shunt of coronary arteries, stroke, claudication, stent/shunt of leg arteries), chronic kidney disease stage IV–V, severe visual disturbances, severe nonproliferative or progressing proliferative retinopathy, macular edema, severe muscular‐skeletal diseases of legs or severe peripheral neuropathy, disabling somatic, or psychiatric disease.

Of the 78 initially screened patients, 14 were excluded due to the exclusion criteria. Eight patients did not participate after the screening.

### 2.2 Investigations

Fifty‐six subjects eligible for the study performed a VO2 peak test (walking with incremental inclination) on a treadmill (Life Fitness) until volitional fatigue, using a modified Bruce protocol. Participants also donated their blood samples and underwent basic anthropometric and clinical measurements. Blood samples were drawn after fasting at least 8 hr for measurement of glucose, HbA1c, insulin, lipids (triglycerides, total cholesterol, low density lipoprotein, high density lipoproteins). Urine samples were collected in the morning for measurement of albumin/creatinine ratio (measurements were performed in a certified clinical laboratory). Serum for determination of adipocytokines was separated via centrifugation within an hour after blood was taken, aliquoted, and frozen at −80°C before analysis. Samples were processed and stored as previously described (Rovite et al., 2018).

Blood pressure was measured twice after resting for 5‐min in sitting position. Estimated glomerular filtration rate (eGFR) was calculated with the help of Chronic Kidney Disease Epidemiology Collaboration (CKD‐EPI) equation. Body mass index was calculated as weight (kg)/height (m)^2^. After all tests, 56 subjects were randomized to the control (Con, *n* = 26) and interval training (IT, *n* = 30) group. Randomization was performed by a statistician using statistical software R and was aimed at obtaining two groups not differing in basic clinical characteristics (diabetes duration, age, gender distribution, HbA1c, lipids, body mass index, VO2 peak measurement).

The patients in Con group were not controlled until the end of the study. Patients in IT group continued with interval walking training intervention for 4 months.

**FIGURE 1:**
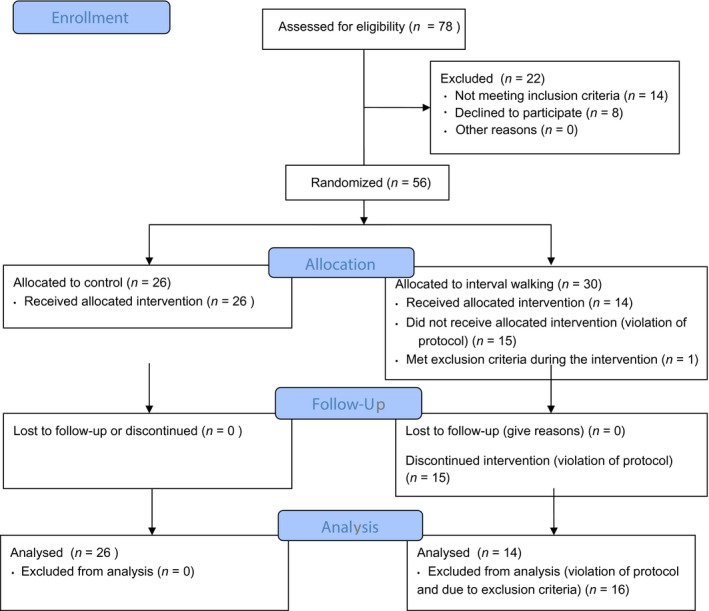
Flow chart representing trial group assignments and completion of intervention

For IT group, criteria for inclusion for final data analysis was set at a priori according to per protocol approach. These criteria were: (a) Accomplished 70%–100% of prescribed training sessions (32–48 sessions *per* intervention). (b) Treatment of type 2 diabetes not changed during the intervention. Thus, at the end of the intervention, 16 patients from IT group were excluded from the analysis (15 patients due number of training sessions 31 and less; one because of initiation of insulin therapy). Thus, 40 patients (Con, *n* = 26; IT, *n* = 14) were included for pre‐ and postanalysis (Supplement 1), Figure 1.

The study was in line with the 1975 Declaration of Helsinki and received approval Nr 1‐03/17 (30.06.2017) of the Scientific Research Ethics Committee of the Institute of Cardiology and Regenerative Medicine of the University of Latvia. All the subjects provided written consent prior to any procedures.

### 2.3 Intervention

**FIGURE 2:**
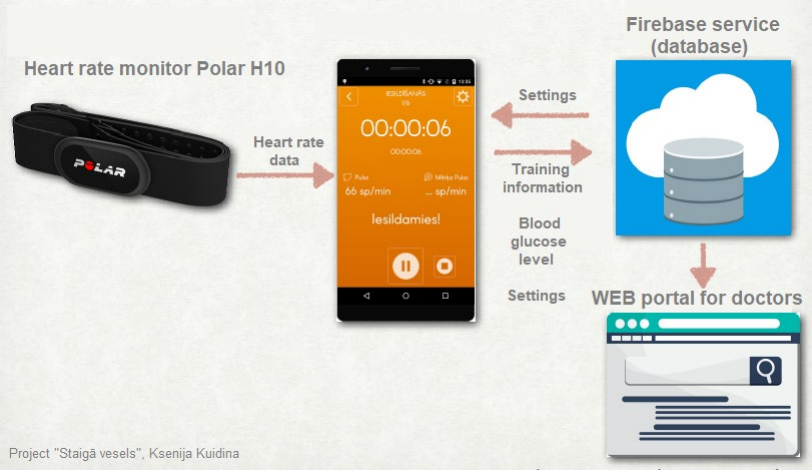
Remote control of adherence to training schedule in interval training group, ensured by data flow among custom application *instawalk*, data storage and project personnel web portal

Subjects in the IT group received mobile smart phones with a custom application *Instawalk*, which delivered a 60‐min training consisting of 3‐min intervals of slow and fast walking with the intensity of 40% and 70% of the peak energy expenditure respectively. The data of VO2 peak test was used for defining the individualized interval walking speeds during slow and fast walking, based on peak energy expenditure rates (Figure 2). Participants had to train three times a week for 4 months. Polar H10 heart rate monitors that were connected to the *Instawalk* application ensured prescribed training intensity level for each individual. Glucose was measured on the training days, before and after the training and before night's rest with glucose meters. The application uploaded heart rate data to the internet data storage, for remote monitoring of training adherence.

Physical exam, blood chemistry analysis, and serum sample collection for determination of adiponectin and leptin (primary outcome measure) were repeated after 4‐months. For IT group, tests were performed within 1–2 weeks after the final training session.

### 2.4 Measurement of leptin and adiponectin in serum

Leptin and adiponectin were measured in serum samples by Luminex technology on *Lincoplex* 200 analyzer using Millipore *MILLIPLEX MAP Human* Adipokine Magnetic Bead Panel 2 kit *HADCYMAG‐61K* (Merck KGaA) and *MILLIPLEX MAP Serum Matrix LHED‐SD*, according to manufacturer's instructions. Leptin and adiponectin concentration was quantitated using the corresponding standard curves (final concentrations for leptin 0, 48, 240, 1,200, 6,000, 30,000, 150,000 pg/ml; final concentrations for adiponectin 0, 26, 128, 630, 3,200, 16,000, 80,000, 400,000 pg/ml), which was performed according to the instructions in the kit. The detection limit of sample was 10–150,000 pg/ml for leptin and 15–400,000 pg/ml for adiponectin. For leptin, intra‐assay coefficient of variation (CV) was <20% CV and interassay CV was <25%, accuracy 90%. For adiponectin, intra‐assay CV was <10%, interassay CV was <15%, accuracy 97%. The measurements were performed in triplicate, and the mean value was used for analysis. The results were recalculated to ng/ml for leptin and µg/ml for adiponectin. Leptin/adiponectin ratio is expressed in ng/µg.

### 2.5 Statistical analysis

Data are presented as mean ± *SD*. Repeated measures ANOVA was performed to analyze the impact of intervention on variables of interest. If the interaction effect was significant, the estimated marginal means were calculated and post‐hoc pairwise comparisons were performed. To adjust for age, gender, diabetes duration, body mass index at baseline, repeated measures ANCOVA method was performed. Pearson correlation coefficient was used for analysis of association between changes (Δ = post–preintervention) of variables of interest. Analysis was done in programme R (R Core Team, 2019, 2019).

## 3 RESULTS

### 3.1 Participants

Clinical characteristics of subjects from Con and IT group from baseline (Pre) and last visit (Post) are presented in Table 1.

**TABLE 1:** Characteristics of study subjects before and after the intervention

	Control (26)		Interval training (14)	
	Pre‐Con	Post‐Con	Pre‐IT	Post‐IT
Gender (male/female)	7/19		6/8	
Age (years)	60.96 ± 8.8		60.64 ± 7.4	
Duration of diabetes (years)	5.73 ± 4.04		8.43 ± 6.97	
Treatment				
Metformin	23		14	
Sulfonylurea	6		5	
DPP4 inhibitors	5		3	
GLP‐1 agonists	1		2	
SGLT‐2 inhibitors	1		1	
VO2 peak (relative, m/k/min)	21.58 ± 4.5	—	23.00 ± 6.61	—
Weight (kg)	88.0 ± 15.2	88.5 ± 14.8	94.6 ± 22.0	92.5 ± 21.6
BMI (kg/m^2^)	32.2 ± 4.92	32.4 ± 5.45	32.6 ± 5.64	31.9 ± 5.89
Waist/hip ratio	0.93 ± 0.07	0.94 ± 0.07	0.97 ± 0.09	0.96 ± 0.08
Total cholesterol (mmol/L)	5.2 ± 1.4	5.3 ± 1.4	5.2 ± 0.7	5.1 ± 0.9
HDL (mmol/L)	1.4 ± 0.3	1.3 ± 0.3	1.3 ± 0.3	1.3 ± 0.3
LDL (mmol/L)	3.3 ± 1.2	3.2 ± 1.3	3.2 ± 0.8	3.1 ± 0.6
Triglycerides (mmol/L)	1.6 ± 0.7	2.4 ± 2.2	2.3 ± 2.8	1.8 ± 0.9
Systolic blood pressure (mmHg)	132 ± 14	132 ± 17	136 ± 18	134 ± 14
Diastolic blood pressure (mmHg)	82 ± 7	85 ± 11	82 ± 11	83 ± 9
HbA1c (%)	6.7 ± 1.1	6.9 ± 1.4	6.9 ± 1.1	6.6 ± 0.7^1^
HbA1c (mmol/mol)	49.7 ± 8.2	51.9 ± 9.8	51.9 ± 8.2	48.6 ± 4.9^1^
Fasting glucose,(mmol/L)	7.3 ± 2.5	7.4 ± 3.2	7.5 ± 2.6	7.3 ± 1.9
HOMA‐IR	4.1 ± 2.1	4.1 ± 2.3	4.7 ± 3.5	5.1 ± 4.8
eGFR (m/ min/ 1.73 m^2^)	109.1 ± 35.1	122.9 ± 50.7	118.9 ± 32.9	134.5 ± 52.3
Albumine/creatinine ratio (urine, mg/mmol)	1.29 ± 3.62	1.99 ± 7.83	3.06 ± 6.05	1.67 ± 3.55^2,^ ^a^
Leptin (ng/ml)	11.73 ± 6.36	12.81 ± 7.05	11.43 ± 9.07	10.59 ± 7.3
Adiponectin (µg/ml)	7.86 ± 3.87	7.49 ± 5.23	7.11 ± 5.74	7.79 ± 3.57
Leptin/adiponectin ratio (ng/µg)	1.85 ± 1.51	2.8 ± 2.28^b^	2.42 ± 3.01	1.38 ± 0.96^3^,^c^

Note

Data are presented as mean ± *SD*. Con, control; IT, interval training. ^1^ *p* = .09, ^2^ *p* = .002, ^3^ *p* = .01 represent *p* for interaction effect after repeated measures ANOVA. No significant changes in *p*‐value was observed after adjustment for gender, age, diabetes duration, BMI at baseline (repeated measures ANCOVA).

^a^ *p* < .001 pre‐IT versus post‐IT.

^b^ *p* = .062 pre‐Con versus post‐Con.

^c^ *p* = .081 pre‐IT versus post‐IT represent *p*‐values for post‐hoc pairwise comparisons.

### 3.2 Compliance

The adherence to training in the IT group was quite low: 14 patients adhered to the protocol and performed 32 and more sessions per study (2–3 times a week). 10 patients performed 20–31 training sessions (1–2 times a week), remaining patients did less than 20 sessions.

### 3.3 Impact of interval walking on albuminuria, glycaemia, lipid profile, insulin sensitivity, and anthropometric measures

**FIGURE 3:**
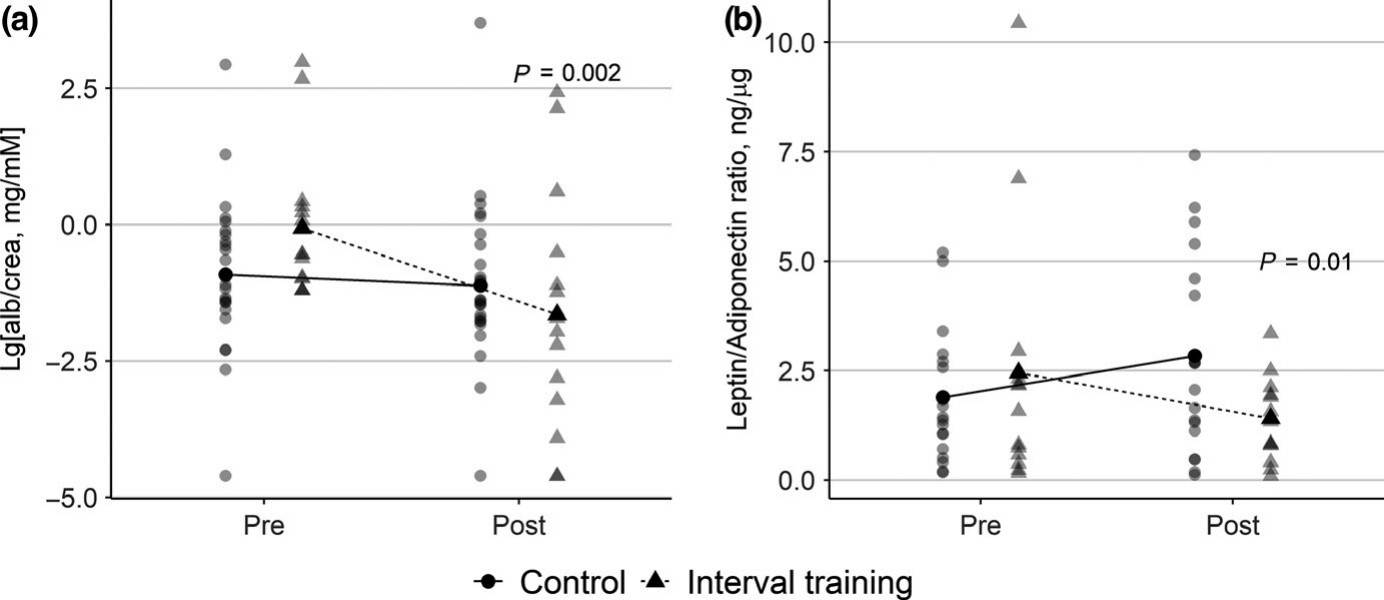
The effects of the intervention on albuminuria and leptin/adiponectin ratio in control and interval training group. (a) Changes in urine albumin/creatinine ratio, expressed as Lg[albumin/creatinine, mg/mmol]. (b) Changes in leptin/adiponectin ratio. Control group (continuous line), interval training group (dotted line). p values on graphs indicate *p* for interaction effect after repeated measures ANOVA

We observed a statistically significant reduction in albuminuria in IT group, compared to Con group (*p* = .002, interaction effect) as a result of the intervention (Figure 3a). HbA1c decreased by 0.3% in IT group and increased by 0.2% in Con group, thus the impact of the intervention on HbA1c was close to statistical significance (*p* = .09 for interaction effect). The intervention did not affect other clinical variables (body composition, lipids, insulin sensitivity, blood pressure etc; Table 1).

### 3.4 Impact of interval walking on adiponectin, leptin, and leptin/adiponectin ratio

Interval walking training did not cause statistically significant changes in leptin and adiponectin level, however, a statistically significant reduction in leptin/adiponectin ratio in IT group in the course of intervention (*p* = .01, interaction effect) was observed (Table 1; Figure 3b).

### 3.5 Correlation between changes in clinical parameters and adipocytokines

When analyzed in the whole cohort (*n* = 50) and in patients of IT group (*n* = 14), changes in leptin/adiponectin ratio (Δleptin/adiponectin) did not correlate with changes in albuminuria (Δalbuminuria). In IT group, Δleptin/adiponectin correlated significantly with changes in hip circumference (*R* = 0.62, *p* = .024), and Δadiponectin (*R* = −0.713, *p* = .006). No correlations were observed between Δleptin/adiponectin ratio and changes in HbA1c, lipids, HOMA‐IR index, BMI, and blood pressure.

## 4 DISCUSSION

The main findings of this study are that the 4‐months of regular interval walking training resulted in a statistically significant decrease in albuminuria and leptin/adiponectin ratio, as well as moderate improvement in HbA1c level in patients with type 2 diabetes. Interestingly, changes in these biomarkers were observed in the absence of significant changes in body composition, serum lipids, and a marker of insulin resistance HOMA‐IR index. This might show that improvement in endothelial function as a result of regular physical activity might emerge before or is not directly associated with clinically significant changes in body composition and blood biochemistry parameters.

The mechanisms by which physical activity might exert its positive effects on vasculature include adaptations of blood vessels to hemodynamic effects of training, reduction in insulin resistance, and improved oxygen supply (Green et al., 2017). It is known that vascular insulin resistance, characteristic for type 2 diabetes, leads to nitric‐oxide deficiency and overproduction of endothelin‐1, contributing to vascular damage. Physical activity reduces vascular insulin resistance (Ahmed, Blaha, Nasir, Rivera, & Blumenthal, 2012). In our study, we did not observe changes in HOMA‐IR, a marker of insulin resistance, in IT group. However, the decrease in leptin/adiponectin ratio in IT group confirms reduction in insulin resistance (López‐Jaramillo et al., 2014). We propose that the lack of the effect of IT on HOMA‐IR could be explained by hepatic and peripheral clearance of insulin (Chang et al., 2006; Kim et al., 2016).

The decrease in albuminuria in IT group could be explained by the beneficial vascular effects of interval walking training. Physical activity improves endothelial function through amelioration of phosphatidylinositol‐3‐kinase/protein B (Akt) pathway (Mitranun, Deerochanawong, Tanaka, & Suksom, 2014; Naylor et al., 2016). Regular training modifies blood flow, luminal shear stress, arterial pressure, and oxygen supply, which have been shown to translate into the modification of processes of atherosclerosis, including vascular permeability (Green et al., 2017). We suppose that albuminuria could be used in clinical practice as a biomarker of cardioprotective effects of physical training for patient motivation. Promising data about the effects of physical activity on progression of complications of diabetes have been obtained previously (Pongrac Barlovic et al., 2019; Tikkanen‐Dolenc et al., 2017).

Interval walking training decreased leptin/adiponectin ratio in the absence of significant changes of BMI, waist and hip circumference in IT group. However, changes in leptin/adiponectin ratio significantly correlated with changes in hip circumference in IT group. Unfortunately, we did not measure fat and lean mass in our participants before and after the intervention, so we do not know if interval walking had affected these parameters. Higher physical activity is associated with lower fat mass in the presence of the same BMI (Bradbury, Guo, Cairns, Armstrong, & Key, 2017). In addition, caloric deficit in IT group due to increased physical activity could lead to a reduction in circulating leptin levels, due to the sensitivity of this parameter to energy balance (Becic et al., 2018). Finally, exercise can decrease leptin resistance, a condition characteristic for type 2 diabetes and contributing to hyperleptinaemia (Dyck, 2005) and thus lead to decrease in leptin levels.

The lack of correlation between Δleptin/adiponectin ratio and Δalbuminuria in IT group indicated that these beneficial effects of interval training were not directly associated in our study. It is possible that leptin/adiponectin ratio and albuminuria represent different pathophysiologic processes of complications of diabetes, not associated directly the early stages of vascular damage (Forman, Fisher, Schopick, & Curhan, 2008). However, some studies indicated association between leptin and adiponectin levels and vascular complications of diabetes associated with albuminuria (Katsiki et al., 2018; Lim et al., 2015; López‐Jaramillo et al., 2014).

Our study has demonstrated low compliance with the physical activity plan even when supported by mobile technology. The mobile application *instawalk* was aimed at providing a personalized and safe interval walking training; however, the application did not send patients notifications/reminders. In patients who were compliant with the training plan, a positive effect on HbA1c which was close to statistical significance was observed. Our results stress the inertia of T2D patients to change their lifestyle, and underline the necessity to integrate solutions for improved motivation and compliance into similar apps in the future.

The main limitations of our study are lack of dietary counseling and control for energy consumption, lack of interterm VO2 peak testing to adjust for improvements of physical fitness in the course of the intervention, and lack of body composition measurements. These limitations might be the main explanation for the absence of a statistically significant effect of the intervention on glycaemic control and anthropometric measures (BMI, waist and hip ratio).

In conclusion, this study was performed to test the hypothesis that 4 months long interval walking training managed through smart mobile devices affects molecular mechanisms of vascular complications of diabetes. We report, that interval walking statistically significantly reduced albuminuria and leptin/adiponectin ratio, and had a moderate positive effect on HbA1c in patients with type 2 diabetes. As albuminuria, leptin/adiponectin ratio and glycaemic control have been associated with progression of vascular complications of diabetes, our results prove that interval walking training is effective for improvement of vascular health in diabetes. Albuminuria could be used in clinical practice as a simple biomarker of cardioprotective effects of physical training. Finally, solutions to foster compliance of patients with the physical activity plan are needed.

## CONFLICT OF INTEREST

The authors have no conflict of interest to declare.

## AUTHOR CONTRIBUTIONS

J. S. and V.P designed the clinical set‐up of the study. J.S. wrote the manuscript. K.O. was involved in coordination and technical support to patients and collection of data. K.K. and A.C. were involved in design of the smart phone application *Instawalk*, technical support and data transfer. I.F. was involved in recruitment of patients. G.V. did the measurement of adipocytokine concentration. L.P. and J.V. were responsible for statistical analysis. A.K. and L.S. designed the study and edited the manuscript.
